# Physiological renormalization using systems therapeutics

**DOI:** 10.2144/fsoa-2019-0106

**Published:** 2019-10-31

**Authors:** Greg Maguire

**Affiliations:** 1 BioRegenerative Sciences Inc, and NeoGenesis Inc, San Diego, CA, USA

**Keywords:** checkpoint inhibitors, personalized medicine, physiological renormalization, systems therapeutics

A number of new therapeutics in development and in the marketplace, including ‘checkpoint inhibitors,’ suggest how a paradigm shift in therapeutic strategy is underway for many diseases and indications. The new strategy involves both a renormalization of physiology to treat the disease and a combination of molecule types, working on different pathways, often at different levels and tissues of the system, for example proteins and microbiota, to institute a systems therapeutic approach. This approach had been shown to be efficacious with few adverse side effects.

This article briefly describes and provides examples of a combinatorial strategy for a new means of therapeutic development, a ‘systems therapeutic,’ combined with a new means for disease treatment ‘physiological renormalization.’ The overall idea is to develop systems therapeutics [1], where multiple types of molecules target multiple pathways underlying the disease or condition.

‘Systems therapeutics’ is the use of multiple molecule types to target multiple pathways underlying the indication and is used to develop a state of ‘physiological renormalization,’ the restoration of normal physiology. The idea is to provide diseased tissue with a set of molecules found in the normal state in order to restore normal physiology [2]. Thus, using a new strategy that departs from reductionism and targeted approaches, a transformational platform technology is used where systems therapeutics institutes physiological renormalization. In one example of the strategy, multiple molecule types from stem cells and other cell types of normal tissues are used to return diseased tissues to a normal state [2]. In Maguire *et al.* [2], the secretome from four different stem and other support cell types, constituting the systems therapeutic, was used to rescue neurons through physiological renormalization. Because 70–90% of disease risk is associated with our exposome [3,4] and does not involve the genome, much of the strategy is focused on physiological renormalization of fast, directly coupled protein circuits. These protein circuits are susceptible to environmental perturbations that may cause self-templating prion-like proteins, leading to spreading dysfunction, as found in prion diseases [5]. Even if systems biology is used to develop targeted therapeutics, the end result is still antediluvian; diseases are multifactorial and require systems therapeutics [6].

A physiological renormalization strategy has been successfully used to develop ‘checkpoint inhibitors’ that reset the adaptive immune system so T cells can once again attack cancer cells [7], a strategy and technology for which the 2018 Nobel Prize in Physiology or Medicine was awarded to James Allison, PhD. This immune system renormalization strategy departed from two basic strategies of the past in which: the immune system was upregulated to induce enhanced attack of the cancer cells, or toxins were used to directly attack the cancer cells. Both previous strategies caused collateral damage to host tissue, along with the intended destruction of tumor cells. The physiological renormalization induced by the checkpoint inhibitor can be extended using a systems therapeutics approach; where along with the checkpoint inhibitor, the patient can also be fed fiber to better enable the checkpoint inhibitor's efficacy [8]. T cell function on cancer cells may be more efficacious under checkpoint inhibition because consumption of fiber has been shown to boost T cell metabolism [9]. Thus, a combined action at multiple pathways and at multiple levels of the system using a systems therapeutics approach, enhances physiological renormalization, helping to quell the cancer.

Systems therapeutics for physiological renormalization was also successfully used by Mao-Qiang *et al.* [10] to develop a commercialized topical product containing multiple lipid types to mimic and restore barrier function of the skin's stratum corneum. As a result of restored barrier function, a reduction in skin and systemic inflammation associated with aged and other inflammatory conditions of the skin was found [11]. In this case, cholesterol, free fatty acids and ceramide are combined in a topical emollient that feeds the necessary lipids to keratinocytes so that multilamellar bodies are produced and excreted by the keratinocytes to rebuild the stratum corneum. The stratum corneum is responsible for the skin's barrier function that prevents moisture loss and protects from environmental insult. Dysfunction of the stratum corneum is associated with dermatosis, including atopic dermatitis [12] and may lead to an induction of chronic systemic inflammation [11], a causal factor in many diseases.

Systems therapeutics for physiological renormalization is under development to treat neurodegenerative disease [2], where the multiple types of molecules from stem cells and other cell types that surround and support neurons, was applied to damaged neurons for their rescue. Here again the approach is to use multiple molecule types at multiple levels of the system. This approach can quench the prion-like spread of misfolded, dysfunctional proteins and re-establish the matrix surrounding neurons so that endogenous signaling molecules from surrounding cells can use the matrix to shuttle from healthy cells to degenerating cells for purposes of neuronal rescue.

At the level of protein pathways, many natural molecular, cellular and tissue functions are initiated and maintained by protein level circuits. For example, caspase-mediated programmed cell death – that is, apoptosis – is orchestrated by a circuit of proteases that activate one another through cleavage [13]. A number of advantages are offered by targeting protein circuits offers as compared with targeting genetic circuits. These include quicker operation, direct coupling to endogenous pathways, single transcript delivery and function without genomic integration [14]. As such, therapeutics at the protein level could be very important for neurodegenerative diseases as the disease state may often occur at the protein level, not the genomic level [2]. Although genomic correlates have been demonstrated in an animal model of amyotrophic lateral sclerosis, transcripts were normal, suggesting that the disease state occurs at the level of protein translation or post-translation despite the association of some genetic defects [15].

Many diseases, if not most, are a function of our exposome and not our genome, affecting many nongenomic pathways [4]. The fact that many nongenomic pathways are affected in diseases argues for using systems therapeutics to target those pathways. This may be a preferred strategy compared with targeted approaches that affect only one pathway, often doing so at the irrelevant target level of the genome. Using the systems therapeutic to institute physiological renormalization for disease remediation has proven efficacious for a number of disease states [2,7,8,11]. The therapeutic strategy using systems therapeutics, especially when the multiple molecule types are bioidentical molecules, to induce physiological renormalization has been shown to be efficacious with few adverse side effects. This promising new therapeutic strategy, for which there are multiple lines of evidence [2,7,8,11], could have far-reaching implications for chronic diseases and indications and as a new means for therapeutic development.
